# Metabolomics and eicosanoid analysis identified serum biomarkers for distinguishing hepatocellular carcinoma from hepatitis B virus-related cirrhosis

**DOI:** 10.18632/oncotarget.19173

**Published:** 2017-07-10

**Authors:** Zhi-Gang Gong, Weijie Zhao, Jianbing Zhang, Xi Wu, Jing Hu, Guo-Chang Yin, Yong-Jiang Xu

**Affiliations:** ^1^ Key Laboratory of Training, Monitoring and Intervention of Aquatic Sports of General Administration of Sport of China, Faculty of Physical Education, Jiangxi Normal University, Nanchang, China; ^2^ The First Affiliated Hospital of Sun Yat-sen University, Guangdong, China; ^3^ Shanghai Institutes for Biological Sciences, Chinese Academy of Sciences, Shanghai, China; ^4^ Department of Medicine, University of California San Diego, La Jolla, California, United States

**Keywords:** metabolomics, hepatocellular carcinoma, eicosanoid, biomarker, liver cirrhosis

## Abstract

Hepatocellular carcinoma (HCC) is one of the most common cancers in the world. It is a type of inflammation-related cancer that usually follows liver hepatitis that mostly caused by hepatitis B virus (HBV) in China. However, the metabolism disturbance of HCC and HBV-cirrhosis is not yet fully understood. In addition, there is little research on the relationships between inflammation mediators and HCC. In this study, we investigated serum metabolic abnormalities in HBV-cirrhosis and HCC patients through non-targeted metabolomics and targeted eicosanoid analysis. Metabolomic analysis identified 14 metabolites, *i.e*. malate, citrate, succinate, lysine, carnitine, proline, ornithine, serine, phenylalanine, tyrosine, arachidonic acid arabinose, galactose and uric acid are consistently altered in HBV-cirrhosis and HCC patients. Meanwhile, eicosanoid analysis uncovered several prostaglandins and leukotrienes are implicated in pathological processes in HBV-cirrhosis and HCC. Finally, these identified biomarkers possessed strong potential to distinguish and diagnose HCC from healthy controls and HBV-cirrhosis patients. This study provided a new perspective to understand the mechanism and discover probable biomarkers of HCC.

## INTRODUCTION

Hepatocellular carcinoma (HCC) is one of the most common malignant tumors; over 630,000 new cases have been diagnosed each year [1]. More than 80% of HCC cases are developed from liver cirrhosis that associated with chronic inflammation, such as the infection of hepatitis B or C virus (HBV or HCV) and non-alcoholic steatohepatitis (NASH) [2–4]. HBV-related liver cirrhosis has been epidemiologically linked to the development of HCC for more than 30 years [5]. Despite our increasing the understanding of pathogenesis of HCC [6], the metabolic changes of HBV-cirrhosis and HCC are not completely known. On the other hand, there are huge demands of reliable and robust biomarkers for clinical diagnosis of HCC. Although alpha-fetoprotein (AFP) has widely been used for HCC diagnosis [7], it is not perfect because of its high false positive and false negative ratio [8]. Therefore, it is imperative to develop more sensitive and specific markers for HCC diagnosis.

Eicosanoids, including prostaglandins and leukotrienes, are biological active lipids that have been implicated in various pathological processes, such as asthma, diabetes, cardiovascular disease and cancer [9, 10]. The investigation of eicosanoids can discover a missing link between inflammation and cancer, and then help to understand the underlying molecular mechanisms of cancer progression [11].

Metabolomics is a powerful tool in disease mechanism investigation, which provides abundant information for biomarker discovery, pathogenesis, and personalized treatment [12]. Recently, great efforts in metabolomics have been made in searching for HCC markers, with some metabolites being found as prospective biomarkers [13–15]. However, few of these studies concerned the distinction between HBV-cirrhosis and HCC [16]. In addition, the study on the role of eicosanoids in HBV-cirrhosis and HCC is absent.

In this study, we investigated the serum sample of HBV-cirrhosis and HCC patients using non-targeted metabolomics and targeted eicosanoid analysis. Several metabolites and eicosanoids were identified to be associated with the distinguishing HBV-cirrhosis and hepatocellular carcinoma. These compounds were further evaluated their diagnostic and prognostic potential biomarkers for hepatocellular carcinoma.

## RESULTS

### Clinical patient characteristics

Clinical characteristics of patients and healthy subjects are detailed in Table 1. For hepatocellular carcinoma patients, serum concentrations of AFP, alanine transaminase (ALT) and aspartate transaminase (AST) were used to diagnose and indicate the extent of liver damage. For HBV-cirrhosis patient, serum ALT levels and HBsAg were used to character hepatitis B virus infection and liver injury.

**Table 1:** Demographic and clinical characteristics of the patients

Patients characteristics	Healthy controls (n=39)	HBV-cirrhosis (n=49)	HCC (n=51)
Age	50.3±7.5	52.3±8.7	56.1±10.3
Gender (F/M)	20/19	28/21	31/20
BMI	28.5	26.7	24.6
ALT (U/L)	8-15	48.0 (15-342)	54.0 (17-453)
AST (U/L)	8-15	56.0 (17-440)	64.0 (21-542)
AFP	0	35.7 ± 87.3	233.9 ± 430.3
AFP>20 (ng/mL)	0	10 (39)	37 (14)
HBsAg	-	+	+

### Non-targeted metabolomics

In order to obtain high-quality data, technical errors originated from sample preparation and instrumental analysis should be eliminated. In this study, quality control (QC) samples were used to evaluate the stability of the method. Principal component analysis (PCA) showed that QC samples are clustered together, suggesting this method has good stability and reproducibility (Supplementary Figure 1).

**Figure 1:**
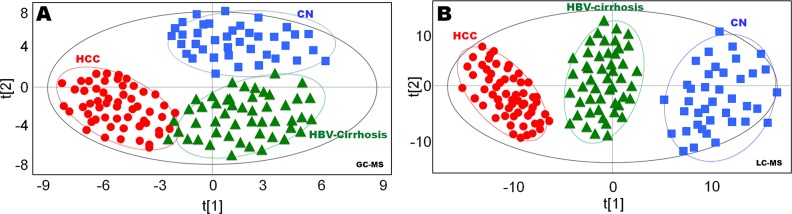
Statistical discrimination of healthy controls (CN), hepatitis B virus cirrhosis (HBV-cirrhosis) and hepatocellular carcinoma (HCC) patients **(A)** Orthogonal projections to latent structures discriminant analysis (OPLS-DA) score plot obtained from gas chromatography (GC)-mass spectrometry (MS) data; **(B)** OPLS-DA score plot obtained from liquid chromatography (LC)-MS data. The x axis, t [1], and y axis, t [2], indicate the first and second principle components, respectively.

Multivariate statistical analysis was performed on all features acquired from both GC-MS and LC-MS analysis. In OPLS-DA plots, GC-MS data showed clear separation of hepatopaths from healthy controls (Figure 1A), LC-MS data also demonstrated similar distinct separation (Figure 1B), with strong modeling fit R^2^Y values of 0.891 (GC-MS) and 0.913 (LC-MS), as well as good prediction Q^2^ values of 0.783 (GC-MS) and 0.812 (LC-MS). To validate the reliability of the prediction model, permutation test (n = 20) was calculated (Figure 1B). The Q^2^-intercept value (−0.192) of the prediction model was lower than 0.05, indicating that the model is statistically sound, and that its high predictability is not due to over-fitting of the data (Supplementary Figure 2).

**Figure 2:**
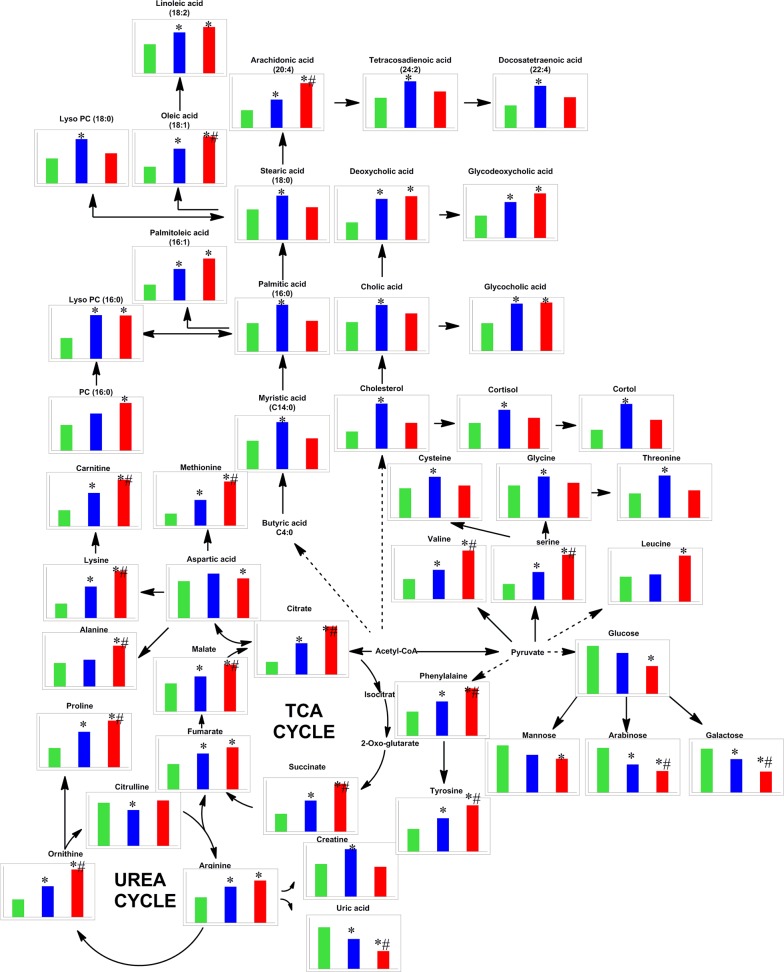
Metabolic network of the relevant metabolites in this study The normalized levels of each metabolite in healthy subjects (CN, green column), hepatitis B virus cirrhosis (HBV-cirrhosis, blue column) and hepatocellular carcinoma (HCC, red column) are shown under the chemical name. * *p* < 0.05, HBV-cirrhosis *vs* CN; #*p* < 0.05, HCC *vs* HBV-cirrhosis.

**Figure 3:**
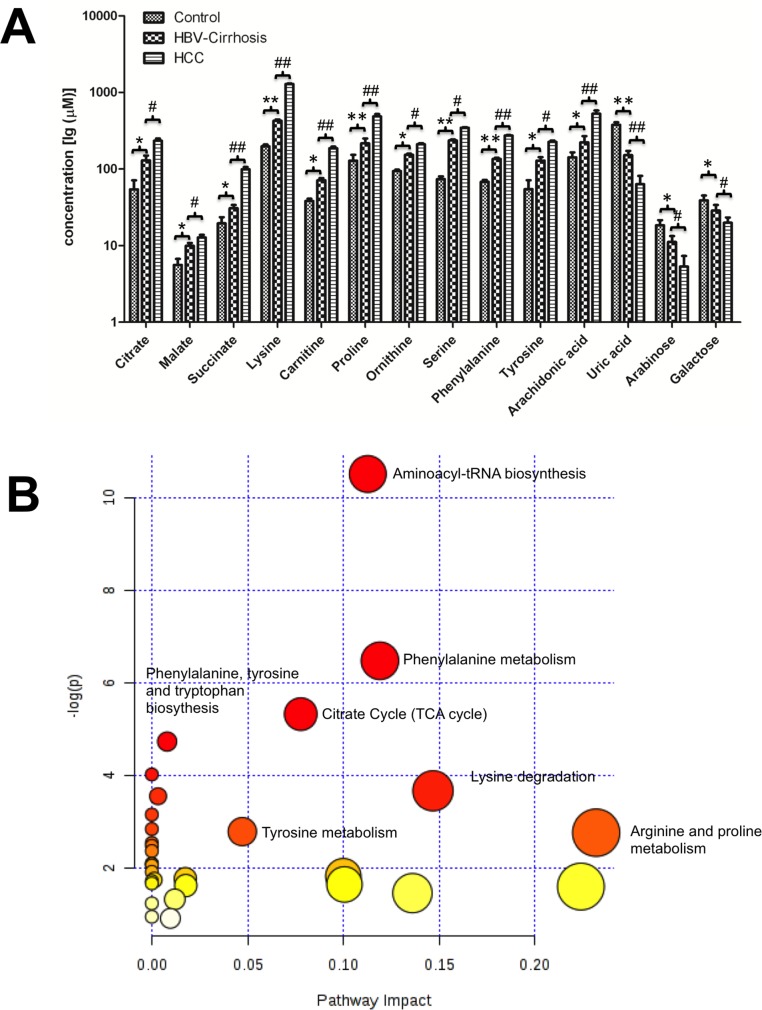
The metabolic changes related to the pathology of hepatitis B virus cirrhosis (HBV-cirrhosis) and hepatocellular carcinoma (HCC) patients **(A)** The serum concentrations of 14 potential biomarkers in all groups. * *p* < 0.05, ** *p* < 0.01, HBV-cirrhosis *vs* healthy control (CN); # *p* < 0.05, ## *p* < 0.01, HCC *vs* HBV-cirrhosis. **(B)** The summary of the involved pathways for these aberrant metabolites in the carcinogenesis and the development of hepatocellular carcinoma. All matched pathways were shown according to *p* values from the pathway enrichment analysis (y-axis) and pathway impact values from pathway topology analysis (x-axis), with the most impacted pathways colored in red.

Based on OPLS-DA models, pair-wise comparisons were carried on healthy controls, HBV-cirrhosis and HCC patients respectively (Supplementary Figure 3). It indicated that 427, 382 and 243 mass spectrometry features significantly changed between HBV-cirrhosis and control, HCC and control as well as HCC and HBV correspondingly (VIP > 1.0 and *p* <0.05). Subsequently, 42 metabolites were identified to be strongly associated with liver cirrhosis, including elevated fatty acids, glycine, serine, malic acid, succinic acid, valine and bile acids, together with downgraded carbohydrates, creatine and uric acid. On the other hand, 31 perturbed metabolites were highly correlated with the presence with HCC, involving in amino acid metabolism, lipid metabolism, acylcarnitine metabolism and energy metabolism (Figure 2). Among these metabolites, 14 metabolites were stepwise altered in HBV-cirrhosis and HCC, which may be potential biomarkers of hepatocarcinogensis. Therefore, these metabolites were used as a panel of biomarkers to diagnose HCC from healthy control and HBV-cirrhosis groups, including remarkably elevated malate, citrate, succinate, lysine, carnitine, proline, ornithine, serine, phenylalanine, tyrosine, arachidonic acid and decreased arabinose, galactose and uric acid. These biomarkers were further determined their concentrations based on standards (shown in Figure 3A).

The biologic pathways that involved in these metabolites and their biological roles were evaluated by enrichment analysis using MetaboAnalyst [17]. A total of nine pathways were found probably associated with liver cirrhosis and carcinogenesis, including aminoacyl-tRNA biosynthesis, phenylalanine metabolism, glutathione metabolism, glyoxylate and dicarboxylate metabolism, alanine, aspartate and glutamate metabolism, the citrate cycle, phenylalanine, tyrosine and tryptophan biosynthesis, glycerolipid metabolism, and glycine, serine and threonine metabolism (Figure 3B).

### Targeted eicosanoid analysis

In eicosanoid analysis, a total of 30 eicosanoids were measured using LC-MS/MS approach. According to Food and Drug Administration (FDA) guidelines, the limit of detection (LOD), accuracy, precision, recovery and stability were validated in this method (Supplementary Tables 2–4).

**Figure 4:**
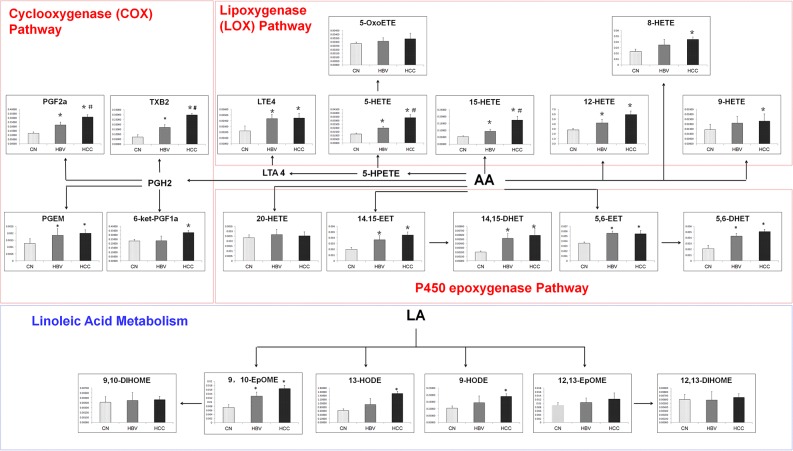
The comparison of levels of metabolites in eicosanoid metabolism pathway *p < 0.05, hepatitis B virus cirrhosis (HBV-cirrhosis) *vs* healthy control; #*p* < 0.05, hepatocellular carcinoma (HCC) *vs* HBV-cirrhosis.

A total of 22 eicosanoids were consistently detected in samples. Among these eicosanoids, prostaglandins, leukotriene E4 (LTE4), thromboxane 2 (TXB2), hydroxyl octadecadienoic acids (HODEs), and most hydroxyeicosatetraenoic acids (HETEs), which are in lipoxygenase (LOX) and cyclooxygenase (COX) pathways, were significantly increased in liver disease groups as compared with healthy group. However, LTE4, PGEM, 12-HETE, 14,15-EET, 14,15-DHETE, 5,6-EET and 5,6-DHETE did not show significant differences between HBV-cirrhosis and HCC group. On the contrary, the serum levels of PGF2α, TXB2, 5-HETE and 15-HETE in HCC group were higher than that in HBV group (Figure 4).

### Comparison of diagnostic potential of metabolites and AFP levels

**Figure 5:**
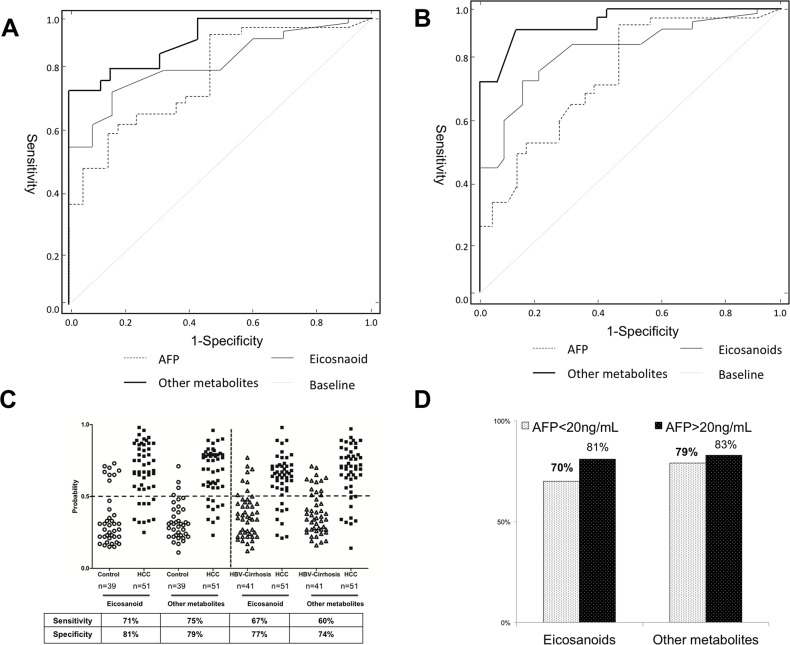
Comparison of ROC curves for combinational of metabolites, or eicosanoids with alpha-fetoprotein (AFP) to diagnose hepatocellular carcinoma (HCC) **(A)** The ROC curve to distinguish HCC patients from healthy controls (CN). **(B)** The ROC curve to distinguish HCC from HBV-cirrhosis patients. **(C)** The discrimination of HCC patients and CN/HBV-cirrhosis subjects using eicosanoids and other metabolites at a cut-off probability of 0.5. **(D)** The diagnostic accuracy of eicosanoids and other metabolites for HCC patients with different concentrations of AFP.

To evaluate the diagnostic effectiveness of these identified biomarkers, the receiver operating characteristic (ROC) curves with the curves with the analysis of differences in the areas under curves (AUC) was employed to estimate the diagnostic accuracy of the pattern metabolites and AFP. In order to ease this examination, four representative eicosanoids (PGF2α, TXB2, 5-HETE and 15-HETE) and other metabolites (lysine, citrate, phenylalanine and serine) in serum were selected as a panel of candidate markers based on their highest VIP and lowest *p* values. In addition, a liner model for the logit function was proposed for estimation of the probability rate of disease diagnosis using concentration of selected metabolites. The logit function was applied to logistic regression method. This function defines the logarithm of odds ratio (logit (*P*)=In *P*/(1-*P*)) and after inverse transformation. The receiver operating characteristic (ROC) curve was plotted using eicosanoids and other metabolites concentrations. The ROC curves based on the multivariable yielded satisfactory results as showed in Figure 5. Using combinational eicosanoids or other metabolites to distinguish HCC subject from the healthy control, the areas under curves (AUCs) were 0.843 (eicosanoids) and 0.886 (other metabolites). These results were slightly better than that of AFP (AUC=0.832). When using combinational eicosanoids and other metabolites to discriminate HCC from HBV-cirrhosis, the AUCs were 0.784 (eicosanoids) and 0.833 (metabolites), which was much better than that of AFP (0.657). At the traditional cut-off value of 0.5 [18], 40 and 41or 39 and 40 of 51 HCC patients were correctly classified when compared with healthy subjects or HBC-cirrhosis patients respectively based on eicosanoids or other metabolites pattern, giving a sensitivity of 71% and 75% or 67% and 70%, as well as a specificity of 81% and 79% or 77% and 74% (shown in Figure 5C). Noteworthy, eicosanoids and other metabolites showed 70% and 79% diagnostic accuracy in these AFP false-negative HCC patients (AFP<20 ng/mL) (Figure 5D).

## DISCUSSION

Metabolomics has been emerged as a useful analytical tool for human disease study, because of its high sensitivity and capability to simultaneously measure many metabolites [19]. Meanwhile, most hepatocellular carcinomas are generally developed from liver cirrhosis, which is primarily caused by chronic hepatitis B virus (HBV) [20]. We hypothesized that liver injure would affect serum metabolites, and could distinguish HBV-cirrhosis and HCC patients. The objective of this study was to investigate the metabolic changes of serum metabolism in HBV-cirrhosis and HCC patients.

Based on non-targeted metabolomic analysis (Supplementary Figure 4), we identified 42 and 31 metabolites are significantly changed in HBV-cirrhosis and HCC patients respectively, when compared with healthy subjects. The further exploration of the relation between these biomarkers was achieved through searching KEGG pathway Database (Figure 2). The result indicated that energy, lipid and amino acid metabolism were affected in the process of HCC.

Subsequently, 14 metabolites were found to be change constantly among healthy control, HBV-cirrhosis and HCC patient, which would be intimately associated with the progression of hepatocarcinogenesis. In principle, the intermediates of tricarboxylic acid (TCA) cycle, such as citrate, succinate and malate were elevated in HBV-cirrhosis patients as compared with healthy subjects, suggesting a strong demand for energy in cirrhosis [21, 22]. Similarly, we noted the highest levels of these intermediates in HCC patients, suggesting the consistent impairment of energy metabolism in hepatocytes might induce oncogenesis[18, 23].

Carbohydrates that are energy sources of hepatocyteshave been reported to change in the serum of liver disease[24–26]. In this study, levels of carbohydrates like mannose, galactose, and arabinose, were substantially declined in HBV-cirrhosis. It is because that carbohydrates would break down into lactate and malate to provide additional energy for hepatic failure from cirrhosis[27]. Furthermore, depletion of carbohydrates could aggravate the inflammation and increase the severity of hepatocyte injury [28]. On the other hand, significant reductions of these carbohydrates were also observed in the serum of HCC patients. The feature of carbohydrates in HCC is consistent with Warburg effect, which is most cancer cells predominantly produce energy at a high rate of glycolysis followed by lactic acid fermentation in the cytosol, rather than through oxidative phosphorylation in mitochondria [29]. Taken together, it suggested dysfunction of energy metabolism might make a great contribution to hepatocirrhosis and hepatocarcinogenesis, appeared in promoting glycolysis and suppressing the TCA cycle.

Several studies have reported dysregulations of amino acids metabolism are associated with hepatic disease and cancer development [15]. Consistent with a previous report [30], significant up-regulation of lysine, proline, ornithine, serine, phenylalanine and tyrosine was found in HBV-cirrhosisand HCC patients. The increased demand of amino acid has been found in malignant tumor cell because of the purpose of tumor protein synthesis and energy demand [30].

Lipid metabolism has also been found to be affected by hepatic disease [31]. We noted elevated levels of fatty acids in patients with liver disease, agreeing with the reported result in a mouse model of nonalcoholic steatohepatitis (NASH) and HCC [32]. The notable increase of fatty acids may have been the result of energy supply and cell membrane synthesis due to aggressive cell proliferation [33]. Interestingly, arachidonic acid was gradually increased in HBV-cirrhosis and HCC patients. Arachidonic acid is a precursor of eicosanoids that are important inflammatory mediators for many diseases [21]. It implied that fatty acids may be involved in the pathology of HCC, since HCC is a type of inflammation related cancer. Besides fatty acids, glycerophospholipids regulate a variety of biological processes including cell proliferation, tumor cell invasiveness, and inflammation [34–36]. Similar to the finding of previous study [37], we observed markedly abnormal glycerophospholipids in patients with hepatic disease, which resulted from rapid membrane PC and PE turnover during liver injury or malignant regeneration [14].

Primary bile acids are products of cholesterol, which are synthesized in liver cell via cytochrome P450-mediated oxidation. Secondary bile acids result from bacterial actions in the colon [38]. They play an important role in the development of liver cirrhosis and cancer, since excessive amount of bile acid produce oxidative stress and DNA damage [39]. As reported in an earlier study [40], aberrations in bile acids, including cholic acid, glycocholic acid, deoxycholic acid and glycodeoxycholic acid were elevated in HBV-cirrhosis, as well as deoxycholic acid and glycodeoxycholic acid in HCC patients, when compared with healthy controls. However, there are no significant differences between HBV-cirrhosis and HCC patients. The finding indicated that bile acids seem to be a sensitive index for hepatocellular dysfunction but not for diagnosis of differential liver diseases [17].

Eicosanoid analysis revealed that serum levels of prostaglandins, such as PGF2α, and PGEM are significantly increased in hepatic disease groups. In particular, PGF2α was noted consistently increase in HBV-cirrhosis and HCC patients. Prostaglandins synthesized by the cyclooxygenase (COX) pathway, act as mediators of inflammatory and immunological reactions [21]. Recent studies demonstrated that high expression COX2 are closely related to carcinogenesis[41]. However, evidence that changes of prostaglandins occur in patients with hepatic cirrhosis remains to be provided. Emerging data indicated leukotrienes that synthesized by lipoxygenases (LOX) from arachidonic acid also play an important role in carcinogenesis[42]. Previous studies have reported LTE4 is increased in patients with colon and prostate cancer [43], and the expression of LTE4 receptors is increased in human pancreatic cancer [44]. Similarly, metabolism of leukotrienes is also impaired in hepatocytes from rats with liver cirrhosis [45]. In addition, others LOX-derived metabolites, such as 8-HETE, 9-HETE, 12-HETE, 13- HODE, and 9-HODE, were also found increased in HBV-cirrhosis and HCC groups, but no appreciable difference between the two. Moreover, the gradual increase of 5-HETE and 15-HETE was observed in HBV-cirrhosis and HCC patients. The results suggested the inflammatory is one of the most important metabolic characteristics of hepatic disease, and which implied the potential application of eicosanoids in the diagnosis of HBV-cirrhosis and HCC.

In clinical diagnosis, there are a strong need for developing an accuracy and effective biomarker to distinguish HCC from liver cirrhosis [46]. Currently, serum AFP level serves as a clinical marker for detection of HCC. However, serum AFP has never been a perfect biomarker for HCC diagnosis, as its low sensitivity and specificity [8]. Therefore, we hypothesized that metabolites concerned with hepatocarcinogenesis would have good diagnostic potential for discrimination and differentiation of HCC and HBV-cirrhosis patients. It is hoped that parallel quantification of these critical metabolites would offer a better diagnostic approach for HCC. In order to evaluate their diagnostic potential, ROC curve was employed for comparing the potential of above-discussed differential metabolites and AFP. The result suggested that parallel investigations of these metabolites exhibited higher sensitivity and specificity to distinguish HCC patients from healthy controls and HBV-cirrhosis patients, when compared with AFP. Therefore, our results suggested that the combination of these metabolites was useful for diagnosis of HCC.

## MATERIALS AND METHODS

### Participants and sample collection

A total of 139 individual serums (49 HBV-cirrhosis patients, 51 HCC patients and 39 healthy subjects) were harvested from the first affiliated hospital, Sun Yat-sen University (Guangzhou, China) between March 2012 and March 2014. All participants voluntarily joined this study, gave written informed consent, and completed a questionnaire that provided demographical information, including age, gender, lifestyle factors, and medical family history. The study protocol was approved by the Institutional Review Boards at the first affiliated hospital, Sun Yat-sen University (FHSYSLC-120203) and was conducted in accordance with the Helsinki Declaration of 1964, as revised in 1975. Whole blood samples were gathered in the morning before breakfast from all participants by venipuncture into untreated tubes and allowed to clot on ice for a maximum of two hours. Serum samples were separated by centrifugation and then stored at −80°C until analysis.

### Sample preparation

Non-targeted Metabolomics analysis: After thawing on ice, the serum specimen (20 μL) was diluted with 100 μL of ice-cold methanol with 10 μg/mL internal standards (listed in Supplementary Table 1). The mixture was shaken vigorously for 30 seconds. After centrifugation at 14,000 rpm for 10 minutes at 4°C, the supernatant fraction was collected and divided into two parts: one (50 μL) for LC-MS analysis and the other one (75 μL) for gas chromatography-mass spectrometry (GC-MS) analysis. For GC-MS analysis, the 75 μL supernatant was dried under nitrogen and derivatized with methoxyamine (50 μg/mL in pyridine) and subsequent trimethylsilylation with MSTFA.

Targeted eicosanoid analysis: Each 20 μL serum was mixed with 180 μL of PBS containing internal standards (listed in Supplementary Table 1). The eicosanoids were extracted using Strata-X 33u Polymeric SPE cartridge (100mg 3mL). Each cartridge was conditioned with 3 mL of MeOH following with 3 mL of H_2_O. After loading, cartridges were washed with 1 ml 10% methanol in water to remove impurities, and the metabolites were then eluted with 1 mL of MeOH and stored at −80°C to prevent metabolite degradation. Prior to analysis, the eluent was dried with N_2_ and dissolved in 50 μl methanol for LC-MS analysis.

### GC-MS and LC-MS analysis

Non-targeted metabolomics: GC-MS analysis was performed on an Agilent 7683B Series Injector (Agilent) coupled to an Agilent 7890A Series Gas Chromatograph System and a 7200 Q-ToF mass detector (Agilent). A fused-silica capillary column HP-5MSI (30 m×0.25 mm i.d., 0.25 μm film thickness) was used. The injector was kept at 250°C. Helium was used as the carrier gas with a constant flow rate of 1 mL/min through the column. The GC oven temperature was maintained at 70°C for 1 min, and then increased to 250°C at a rate of 10°C/min and further increased at 25°C/min to 300°C and held for 6 min. The transfer line temperature was kept at 280°C. Electron impact mode (70 eV) and full scan monitoring (m/z 50 to 550) were used in MS detection. The compounds were identified by comparison of mass spectra and retention time with those of reference standards, and those available in libraries (NIST 0.5, Agilent).

LC-MS analysis for non-targeted metabolomics was performed on a high-performance LC system 1200 (Agilent Technologies, Santa Clara, USA) coupled to a 6520 quadrupole time of flight mass detector equipped with an electrospray ionization (ESI) source. The samples were analyzed in ESI positive and negative ion modes. The separation was performed on an Agilent rapid resolution HT Zorbax SB-C18 column (2.1×50 mm, 1.8 μm) at a column temperature of 50°C. The mobile phases consisted of phase A (water with 0.1 % formic acid) and B (acetonitrile with 0.1 %formic acid). The gradient program was 0-9min, 5-45%B; 9-15 min, 45-100%B; 15-18 min, 100 % B; 18-20 min, 100-5 % B. The flow rate was set at 0.4 mL/min. A 10 μL aliquot of supernatant was injected for each individual analysis. Mass data were collected between m/z 100 and 1000 at a rate of 2 scans/s. The ion spray voltage was set at 4000 V, and the heated capillary temperature was maintained at 350°C. The drying gas and nebulizer nitrogen gas flow rates were 12.0 L/min and 50 psi, respectively. The compounds showing significant differences between samples were searched against the databases of HMDB (Human Metabolome Database), METLIN (www.metlin.scripps.edu) and LipidMaps (www.lipidmaps.org) using mass-to-charge ratio (m/z) and MS/MS fragmentation patterns (Supplementary Table 5). Moreover, available standard samples were utilized for identification and quantitative analysis (Supplementary Table 6).

Targeted eicosanoid analysis was performed on an Agilent 1200 high-performance LC system equipped with a 6495 QQQ mass detector (Waldbronn, Germany). A 20 μL sample of the pretreated sample was injected into LC-MS system and managed by a MassHunter workstation. The column used for the separation was a Waters Acquity BEH RPC18 (2.1 mm × 100 mm, 1.7 μm). The oven temperature was set at 50°C. The gradient elution involved a mobile phase consisting of (A) Acetonitrile/water/acetic acid (60/40/0.02, v/v) and (B) Acetonitrile/Isopropyl alcohol (50/50, v/v). The initial condition was set at 0.1% B. The following solvent gradient was applied: 0-4.0 min, 0.1-55% B; 4.0-4.5 min, 55-99% B; 4.5-5.0 min, 99% B, and the flow rate was set at 0.5 mL/min. The samples were analyzed in ESI-MS in the negative ion mode. The ion spray voltage was set at 3,000 V. The heated capillary temperature was maintained at 350°C. The drying gas and nebulizer nitrogen gas flow rates were 10 L/min and 30 psi, respectively. Eicosanoids were analyzed using scheduled multiple reaction monitoring (MRM). The optimized MRM parameters for eicosanoids were listed in Supplementary Table 2.

### Data processing and statistical analysis

Non-targeted metabolomics data were extracted and aligned using MZmine 2.10. The spectral data were combined into a single matrix by aligning peaks with the same mass and retention time for GC-MS and LC-MS data, respectively. The retention time, mass accuracy and abundance of each peak were normalized to that of the internal standard in each data set. The missing values were replaced with a half of the minimum value found in the data set. Ninety percent filtering was applied to the raw data to include only metabolites that were detectable in 90% of the subjects in at least one of the treatment groups to ensure selection of relevant metabolites. On the other hand, targeted metabolomics data were extracted by MassHunter workstation (Agilent, USA). Concentrations of eicosanoids were calibrated with one of the internal standards.

Multivariate statistical analysis was performed by the SIMCA-P software (version 11.0; Umetrics, Umea, Sweden) and R-studio software, including principal component analysis (PCA) and Orthogonal projections to latent structures discriminant analysis (OPLS-DA). Permutation test was exploited to verify the fitting degree of PLS-DA model. R^2^ represents the explanation capacity of the model, while Q^2^ stands for the predictive capacity of the model. The metabolites with variable importance in the projection (VIP) values larger than 1 in non-targeted metabolomics analysis together with eicosanoids in targeted metabolomics analysis were performed with the Wilcoxon Mann−Whitney test to identify significantly different metabolites,*p* < 0.05 was considered as significant (Supplementary Table 7). Metabolites heatmap of metabolites in non-targeted metabolomics was conducted using MultiExperiment version 4.5.1 (www.tm4.org).

## CONCLUSION

In this study, we performed integrated and comprehensive metabolomics investigations on HBV-cirrhosis and HCC, providing a holistic understanding of the progression of HCC, and identified liver disease-specific potential biomarkers for diagnosis of HCC from HBV-cirrhosis, with an excellent discriminant performance.

## SUPPLEMENTARY MATERIALS FIGURES AND TABLES


