# Supplementary material for: Metabolomics and eicosanoid analysis identified serum biomarkers for distinguishing hepatocellular carcinoma from hepatitis B virus-related cirrhosis
Source: Oncotarget. 2017 Jul 10;8(38):63890–900. doi: 10.18632/oncotarget.19173 (PMC5609970; doi:10.18632/oncotarget.19173)
Supplement: Supplementary file 1 [file oncotarget-08-63890-s001.pdf]

# Metabolomics and eicosanoid analysis identified serum biomarkers for distinguishing hepatocellular carcinoma from hepatitis B virus-related cirrhosis

## SUPPLEMENTARY MATERIALS

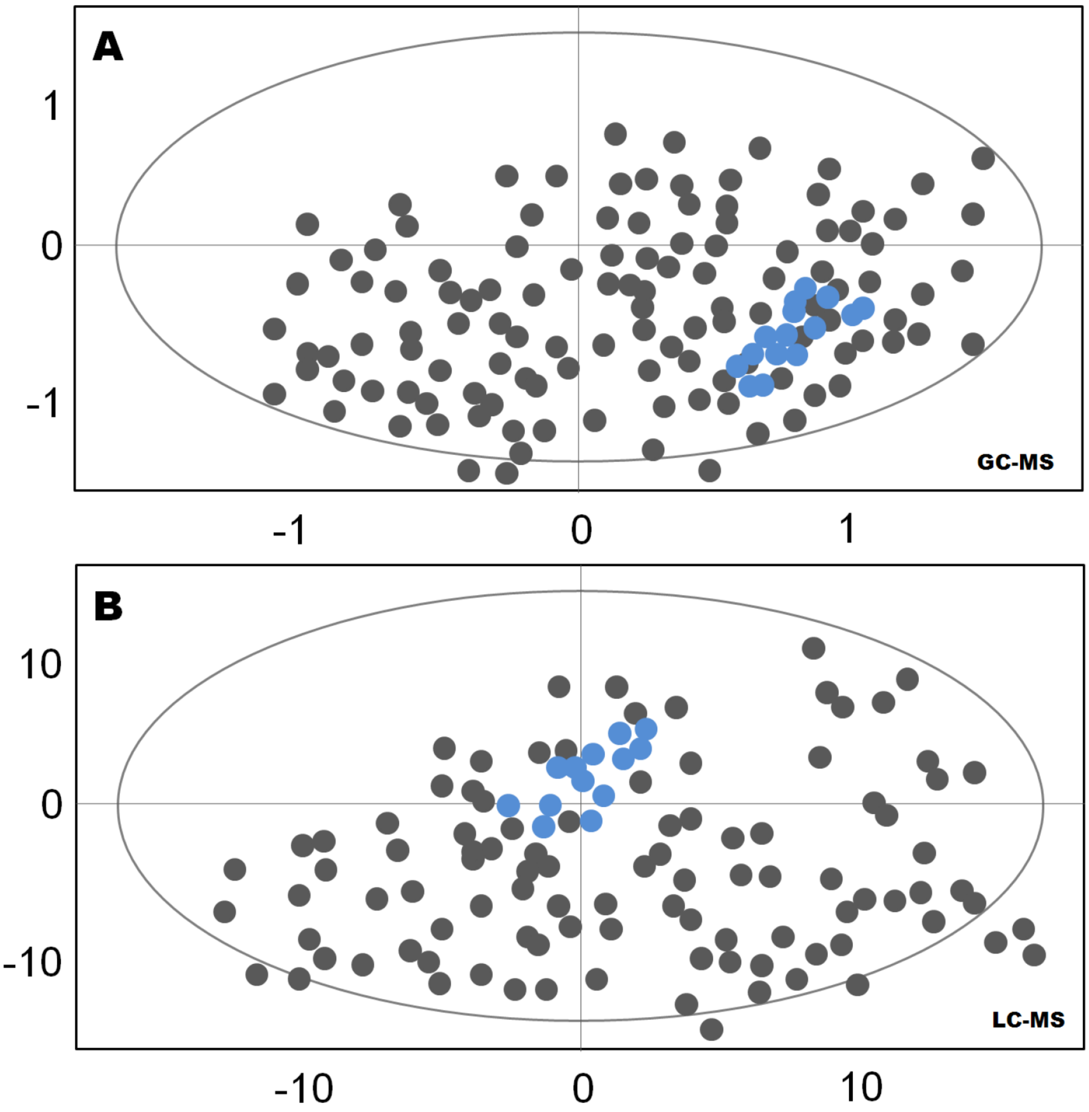


**Supplementary Figure 1: The PCA scores plot for QC sample ( ) and the other samples ( ), showing that the QC samples cluster together.** (**A**) GC-MS; (**B**) LC-MSl.

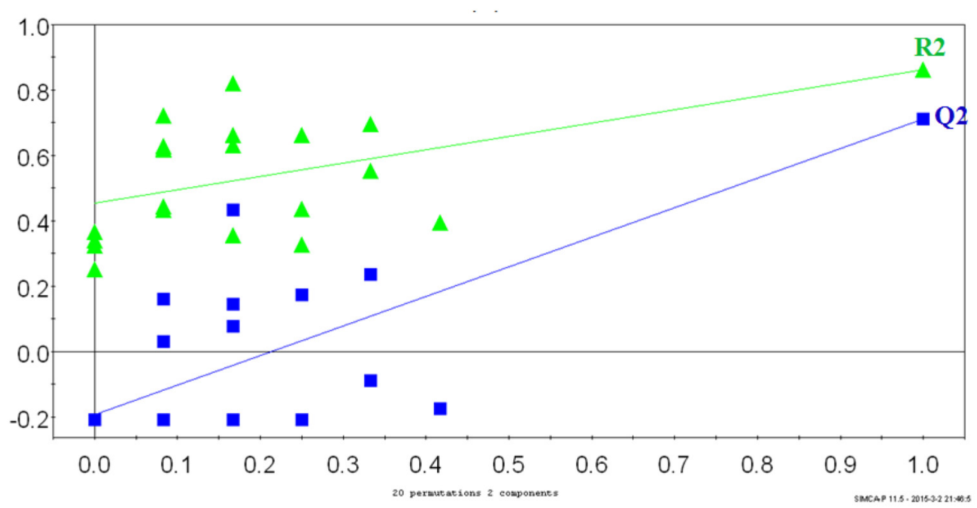


**Supplementary Figure 2: Validation plot obtained from permutation test (n = 20).**

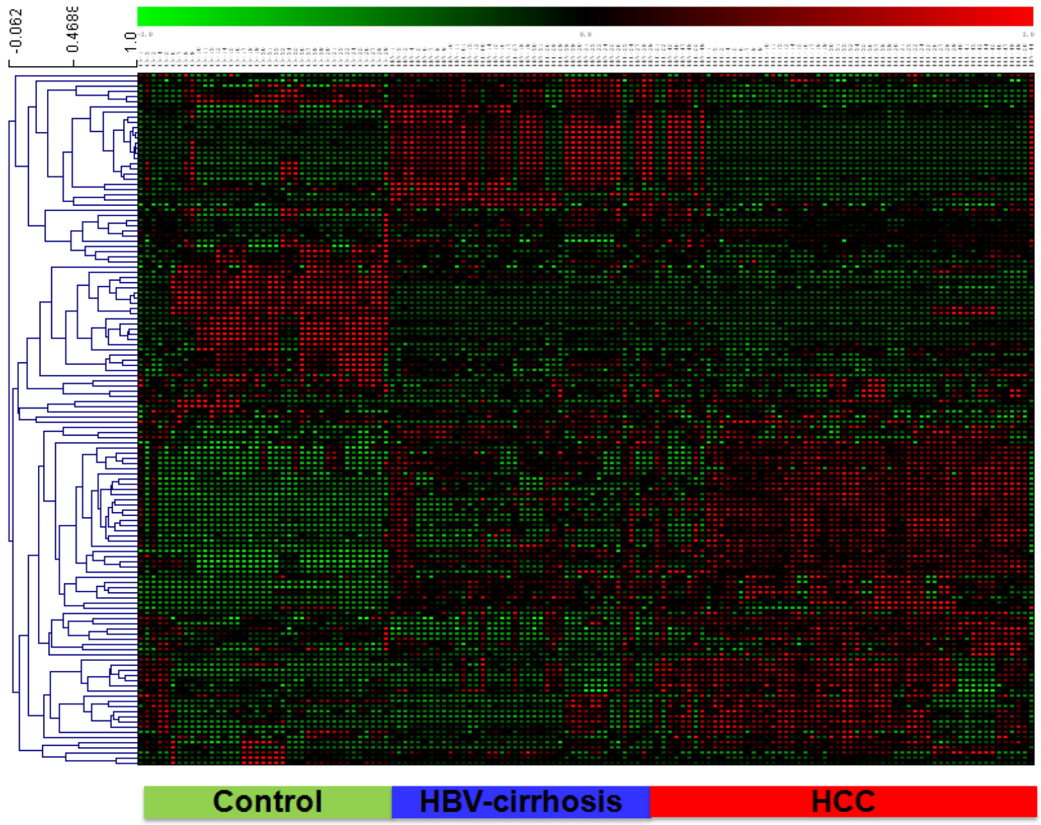


**Supplementary Figure 3: Heat map based on the Pearson distance measure and the Ward cluster algorithm for healthy, HBV-cirrhosis and HCC groups.** Red color indicates high level of metabolites and green color indicates a low level of metabolites, while black means an equal level groups.

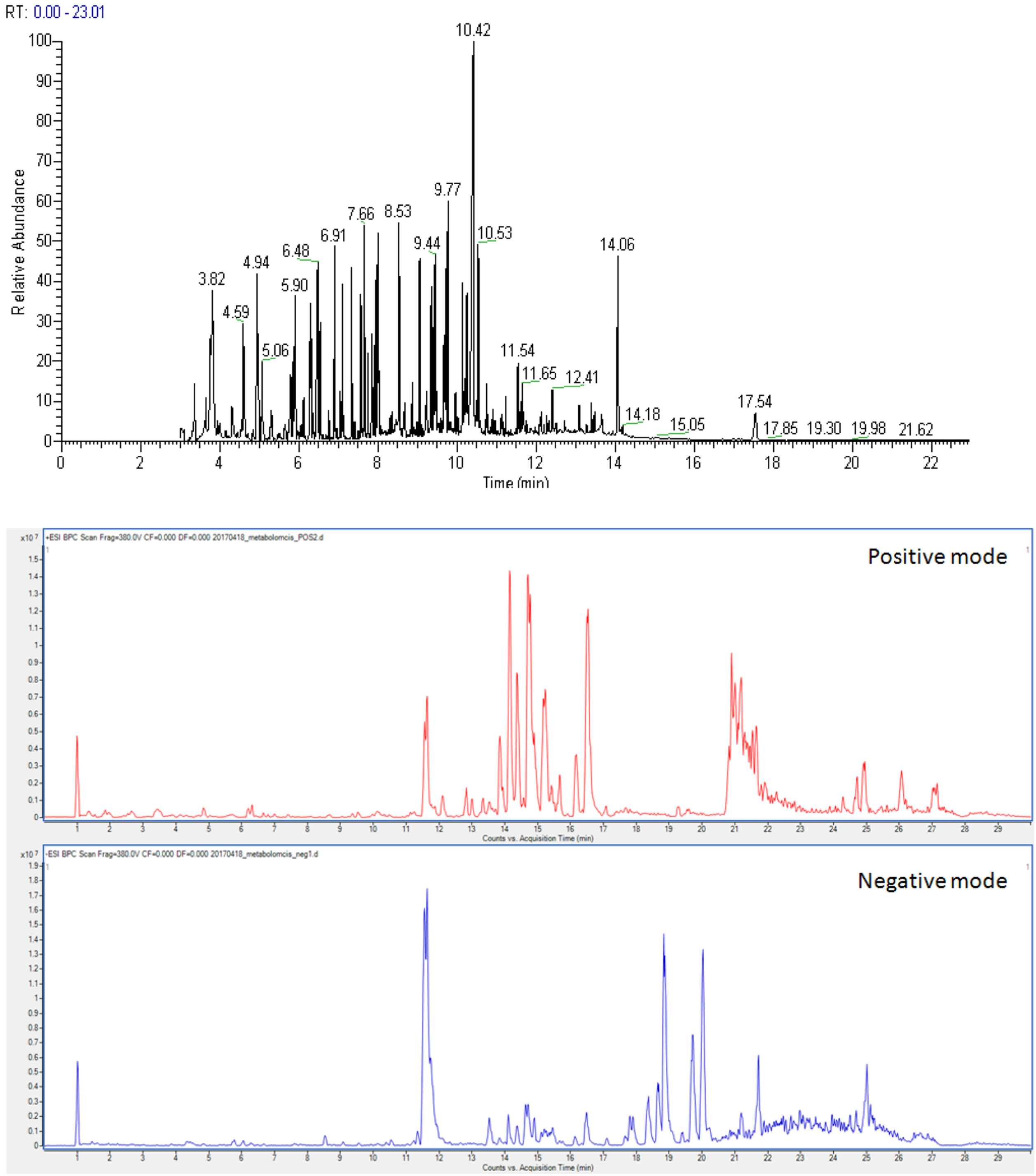


**Supplementary Figure 4: The representative TIC chromatography of GC-MS and LC-MS analysis.**

**Supplementary Table 1: The internal standards used in non-targeted and targeted metabolomics**

| | Internal standards |
|---|---|
| Non-targeted metabolomics | Carnitin C16:0-d3, Cholic acid-d4, chenodexycholic acid-d4, tryptophan-d5, arachidonic acid-d8, Palmitic acid-d3 |
| Targeted metabolomcis | PGF2a-d4; TXB2-d4; 9-HODE-d4; 12,13-EpOME-d4; 15-HETE-d8, 9-HODE-d4, 11,12-EET-d11 |

**Supplementary Table 2: Mass transitions, collision energy, precision and liner range of targeted eicosanoid analysis**

**See Supplementary File 1**

**Supplementary Table 3: Validation of LC-MS assay precision, accuracy and autosampler stability of eicosanoids**

| FAs | Intra-day precision (n = 6) RSD (%) | | | Inter-day precision (n = 6) RSD (%) | | | Accuracy (n = 6) (%) | | 24 hours stability (n =3) |
|---|---|---|---|---|---|---|---|---|---|
| | High | Medium | Low | High | Medium | Low | Intra-day | Inter-day | |
| 6-keto-PGF1α | 2.48 | 1.22 | 1.55 | 1.34 | 7.46 | 2.06 | 99.0 | 113.1 | 100.0 |
| 8-iso-PGF2α | 2.97 | 2.23 | 0.70 | 1.35 | 1.01 | 2.06 | 98.0 | 110.4 | 92.1 |
| PGF2α | 1.82 | 0.96 | 0.79 | 1.24 | 0.91 | 2.24 | 97.6 | 109.1 | 90.3 |
| PGE2 | 1.82 | 0.84 | 0.48 | 0.92 | 1.25 | 2.44 | 97.8 | 107.2 | 93.0 |
| PGD2 | 1.94 | 0.99 | 0.74 | 1.20 | 1.41 | 2.73 | 99.2 | 100.8 | 94.1 |
| TXB2 | 1.93 | 1.28 | 1.36 | 1.47 | 1.31 | 2.70 | 98.3 | 104.0 | 94.5 |
| LTE4 | 2.19 | 1.15 | 0.52 | 1.30 | 1.86 | 2.74 | 99.9 | 96.6 | 94.2 |
| LTB4 | 2.08 | 1.23 | 0.96 | 1.86 | 1.63 | 2.10 | 98.8 | 100.6 | 94.6 |
| 5-HEPE | 2.12 | 1.41 | 3.91 | 1.82 | 2.65 | 5.58 | 98.8 | 100.1 | 94.0 |
| 12-HEPE | 2.80 | 1.09 | 4.26 | 2.71 | 2.06 | 1.61 | 100.9 | 91.5 | 95.5 |
| 15-HEPE | 2.59 | 0.89 | 3.66 | 2.15 | 13.8 | 2.66 | 101.1 | 91.2 | 93.5 |
| 5-HETE | 3.61 | 1.50 | 2.17 | 2.69 | 1.54 | 1.74 | 98.7 | 101.6 | 97.0 |
| 8-HETE | 2.02 | 2.30 | 3.57 | 1.81 | 2.74 | 2.86 | 102.0 | 101.0 | 93.5 |
| 9-HETE | 2.81 | 1.62 | 5.01 | 2.35 | 2.77 | 1.38 | 100.5 | 87.6 | 97.2 |
| 12-HETE | 2.36 | 1.85 | 3.44 | 2.67 | 2.10 | 1.69 | 100.6 | 86.6 | 93.8 |
| 15-HETE | 3.04 | 2.50 | 3.53 | 2.10 | 2.01 | 1.65 | 101.7 | 91.4 | 97.1 |
| 20-HETE | 2.58 | 1.75 | 6.93 | 1.99 | 2.01 | 3.05 | 102.0 | 91.0 | 93.5 |
| 5,6-EET | 2.53 | 2.88 | 3.78 | 2.56 | 1.75 | 1.79 | 100.5 | 90.2 | 95.9 |
| 8,9-EET | 1.44 | 4.09 | 2.40 | 1.57 | 2.01 | 1.33 | 100.6 | 88.4 | 95.6 |
| 11,12-EET | 2.53 | 3.84 | 8.43 | 2.19 | 2.06 | 1.60 | 101.7 | 83.2 | 97.8 |
| 14,15-EET | 2.69 | 2.67 | 6.84 | 2.45 | 2.18 | 1.62 | 101.8 | 83.4 | 96.3 |
| 9-HODE | 2.05 | 1.93 | 2.14 | 2.35 | 2.06 | 1.73 | 101.0 | 89.4 | 96.8 |
| 13-HODE | 2.56 | 1.84 | 4.00 | 1.83 | 1.91 | 1.60 | 101.0 | 90.2 | 98.5 |
| 9,10-DiHOME | 2.33 | 2.18 | 3.48 | 1.97 | 1.46 | 5.43 | 101.6 | 89.7 | 93.5 |
| 12,13-DiHOME | 1.87 | 2.12 | 6.00 | 1.21 | 1.60 | 2.68 | 100.9 | 89.7 | 97.8 |
| 9,10-EpOME | 2.72 | 1.10 | 6.36 | 1.80 | 1.71 | 1.61 | 101.8 | 87.9 | 96.6 |
| 12,13-EpOME | 1.93 | 3.07 | 7.65 | 1.90 | 1.78 | 3.02 | 101.6 | 88.1 | 94.7 |
| 11,12-DiHETrE | 3.22 | 8.17 | 6.23 | 1.46 | 7.76 | 5.36 | 101.9 | 87.3 | 95.7 |
| 14,15-DiHETrE | 8.64 | 1.82 | 5.23 | 4.98 | 1.88 | 3.74 | 101.8 | 94.4 | 96.8 |
| 5-OxoETE | 2.52 | 3.35 | 7.82 | 2.13 | 2.08 | 2.27 | 101.1 | 88.0 | 93.9 |

**Supplementary Table 4: Recoveries of internal standards**

| | Recovery (%) | | |
|---|---|---|---|
| | **High** | **Medium** | **Low** |
| PGF2a-d4 | 71.7 | 66.5 | 68.6 |
| TXB2-d4 | 88.3 | 74.9 | 77.1 |
| 9-HODE-d4 | 94.7 | 69.3 | 72.2 |
| 12,13-EpOME-d4 | 71.8 | 61.5 | 73.2 |
| 15-HETE-d8 | 66.0 | 70.7 | 69.0 |
| 9-HODE-d4 | 90.2 | 68.8 | 65.4 |
| 11,12-EET-d11 | 82.3 | 86.1 | 70.2 |

**Supplementary Table 5: Identification information for LC-MS metabolites**

| Metabolites | RT (min) | MS1 | | MS2 |
|---|---|---|---|---|
| | | Calculated (Da) | Detected (Da) | |
| Positive | | [M+H]+ | [M+H]+ | |
| Glycerol 3-phosphate | 1.1 | 173.0209 | 173.0212 | 151.2, 119.3, 78.9 |
| Malate | 0.8 | 135.0287 | 135.0278 | 99.0, 71.0, 43.1 |
| Creatinine | 1.1 | 114.1251 | 114.1255 | 44.1 |
| Proline | 0.9 | 116.1377 | 116.1370 | 70.2 |
| Ornithine | 1.2 | 133.0970 | 133.0976 | 70.3 |
| Choline | 0.7 | 105.1147 | 105.1141 | 61.2 |
| Carnitine | 1.1 | 162.1124 | 162.1130 | 102.4, 84.5, 59.4 |
| Carnitine (10:0) | 4.5 | 317.2553 | 317.2560 | 102.4, 84.5 |
| Cholesterol | 5.4 | 387.3621 | 387.3624 | 371.3, 369.35 |
| Cortol | 5.5 | 369.2634 | 369.2637 | 351.2, 333.2 |
| Cortisol | 5.9 | 363.2166 | 363.2161 | 345.2, 121.2 |
| MG (14:0) | 10.2 | 303.2529 | 303.2525 | 257.2, 239.1, |
| DG (30:0) | 10.3 | 541.4826 | 541.4831 | 413.2, 352.9, 273.2 |
| DG (32:1) | 10.4 | 567.4982 | 567.4985 | 499.2, 413.2, 301.2 |
| DG (40:4) | 10..6 | 678.6074 | 678.6075 | 619.1, 380.5, 301.2 |
| TG (47:0) | 13.2 | 793.7279 | 793.7276 | 640.2, 542.2 |
| TG (50:2) | 14.3 | 831.7435 | 831.7433 | 651.1, 342.2 |
| LPC (14:0) | 9.7 | 468.3083 | 468.3085 | 400.3, 184.1, 104.1 |
| LPC (16:0) | 10.2 | 496.3397 | 496.3395 | 478.3, 184.1, 104.2 |
| LPC (16:1) | 10.1 | 494.3240 | 494.3243 | 476.2, 184.1, 104.3 |
| LPC (18:0) | 12.3 | 524.3710 | 524.3711 | 506.2, 184.2, 104.1 |
| PC (32:0) | 15.7 | 734.5693 | 734.5689 | 716.2, 184.1, 104.1 |
| PC (40:5) | 16.9 | 836.6163 | 836.6165 | 818.2, 184.2, 104.1 |
| PC (42:2) | 17.3 | 870.6945 | 870.6847 | 852.2, 184.1 |
| PC (30:5) | 17.4 | 808.5850 | 808.5852 | 790.2, 184.1 |
| PC (40:6) | 17.4 | 834.6007 | 834.6010 | 816.2, 184.2, 104.1 |
| PC (42:7) | 17.6 | 860.6163 | 860.6162 | 842.3, 184.1, 104.1 |
| Negative | | $[M-H]^-$ | $[M-H]^-$ | |
| Cholic acid | 5.7 | 407.2803 | 407.2809 | 391.2, 373.3, 345.2 |
| Deoxycholic acid | 5.8 | 391.2854 | 391.2857 | 389.6, 343.3, 327.3 |
| Glycocholic Acid | 5.8 | 464.3018 | 464.3016 | 402.4, 74.5 |
| Glycodeoxycholic acid | 6.2 | 448.3069 | 448.3072 | 330.2, 74.2 |
| PE (32:0) | 16.5 | 690.5079 | 690.5082 | 454.2, 434.2, 255.1 |
| PE (34:1) | 16.2 | 716.5236 | 716.5240 | 452.2, 281.3, 255.2 |

**Supplementary Table 6: Calibration curves and figures of selected biomarkers**

| Metabolites | Regression equation | R2 | LLoQ (µM) | RSD (%) |
|---|---|---|---|---|
| Citrate | y=417.4x−64.5 | 0.995 | 0.3 | 99.5 |
| Malate | y=888.5x−80.4 | 0.999 | 0.3 | 98.9 |
| Succinate | y =964.3x – 277.5 | 0.996 | 0.2 | 99.0 |
| Lysine | y =142.6x – 20.1 | 0.997 | 0.1 | 98.5 |
| Carnitine | y=297.6x+79.4 | 0.996 | 0.36 | 99.1 |
| Proline | y=587x+45.35 | 0.996 | 0.2 | 97.9 |
| Ornithine | y =803.7x – 217.2 | 0.995 | 0.18 | 99.6 |
| Serine | y =197.1x + 46.5 | 0.999 | 0.2 | 98.6 |
| Phenylalanine | y =694.9x – 204.6 | 0.995 | 0.36 | 97.8 |
| Tyrosine | y =646,768x + 12,653 | 0.996 | 0.4 | 98.5 |
| Arachidonic acid | y =124.5x + 30.9 | 0.996 | 0.3 | 98.3 |
| Uric acid | y =923.3x – 11.1 | 0.997 | 0.32 | 96.9 |
| Arabinose | y =515.9x – 36.7 | 0.998 | 0.3 | 98.4 |
| Galactose | y = 602.4x – 62.4 | 0.998 | 0.2 | 98.1 |

**Supplementary Table 7: The identified differential metabolites between HCC, HBV-cirrhosis and control**

**See Supplementary File 2**
